# Supplementary material for: Multiplatform biomarker identification using a data-driven approach enables single-sample classification
Source: BMC Bioinformatics. 2019 Nov 21;20:601. doi: 10.1186/s12859-019-3140-7 (PMC6868758; doi:10.1186/s12859-019-3140-7)
Supplement: Supplementary file 1 — Additional file 1 The file contains eight figures (Figures S1–S8) and four tables (Tables S1-S4) as supplementary results. [file 12859_2019_3140_MOESM1_ESM.pdf]

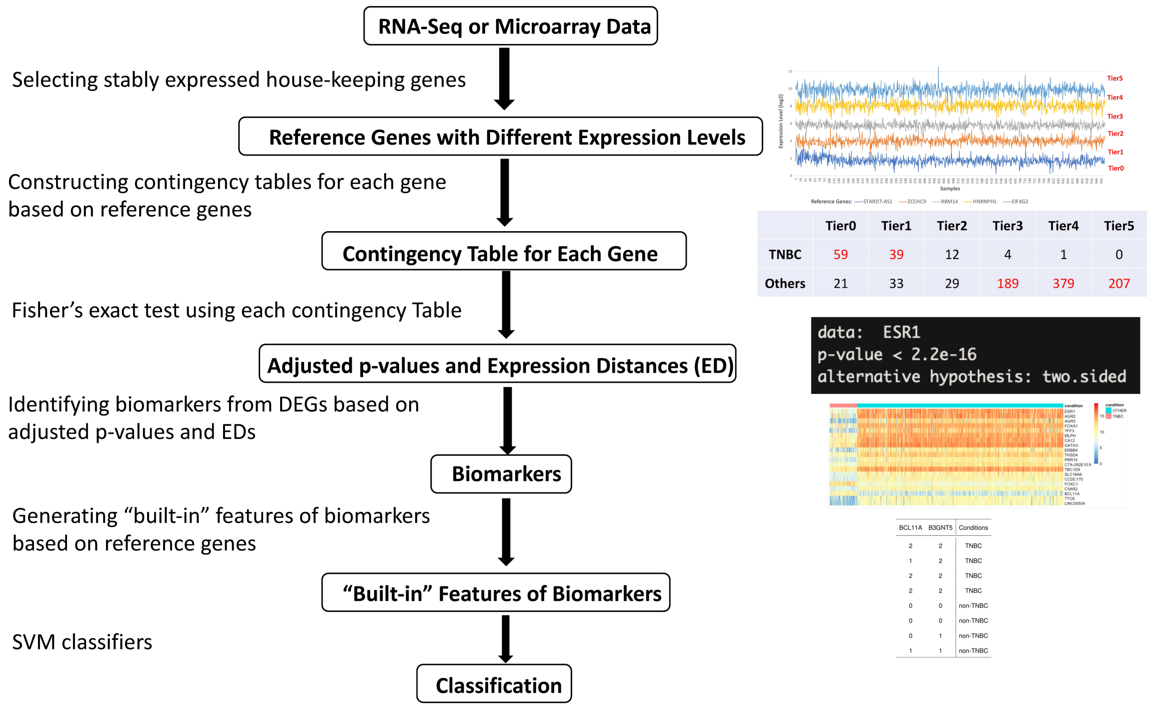

Figure S1: DDR workflow for identification of biomarkers and classification of samples. The results of each step using TCGA-BRCA as example are at the right side.

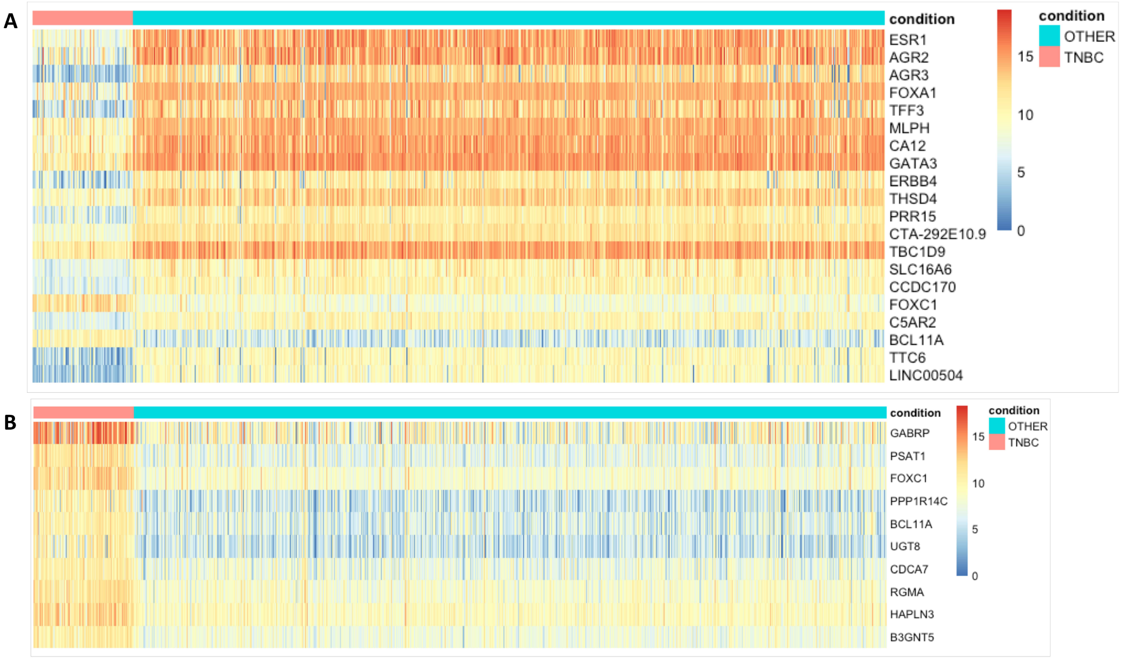

Figure S2: Expression heatmap of top 20 differentially expressed genes between triple-negative breast caner samples (red) and other types of breast cancer samples (turquoise) (**A**) and expression heatmap of top 10 up-regulated genes in triple-negative breast caner samples (red) compared with other types of breast cancer samples (turquoise) (**B**) in TCGA-BRCA RNA-Seq dataset. Expression level of gene is represented as log2(counts+1).

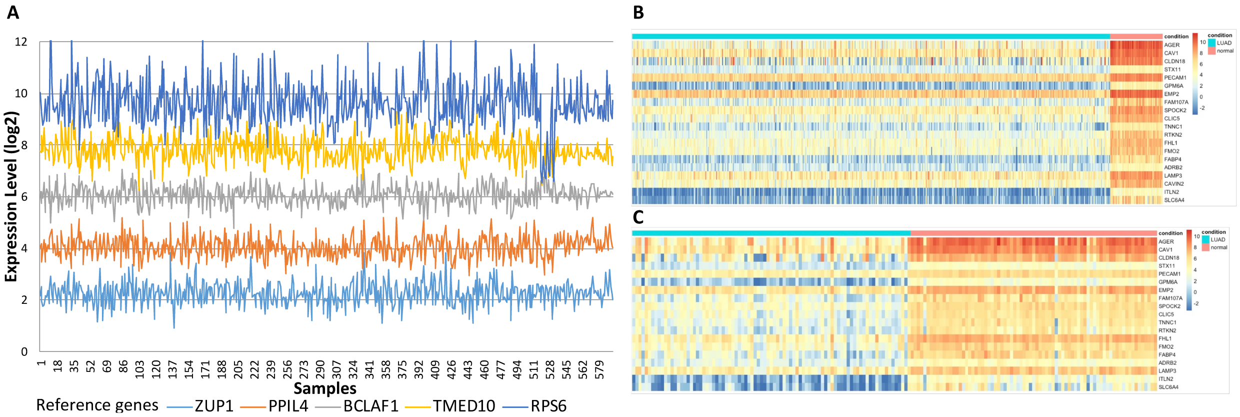

Figure S3: Expression levels of five data-driven reference genes (**A**) and expression heatmap showing top 20 differentially expressed genes between LUAD samples (turquoise) and normal samples (red) (**B**) in TCGA-LUAD RNA-Seq dataset . Expression heatmap of 19 top genes identified from TCGA-LUAD dataset between LUAD samples (turquoise) and normal samples (red) in independent validated dataset (Accession: GSE40419)(**C**).

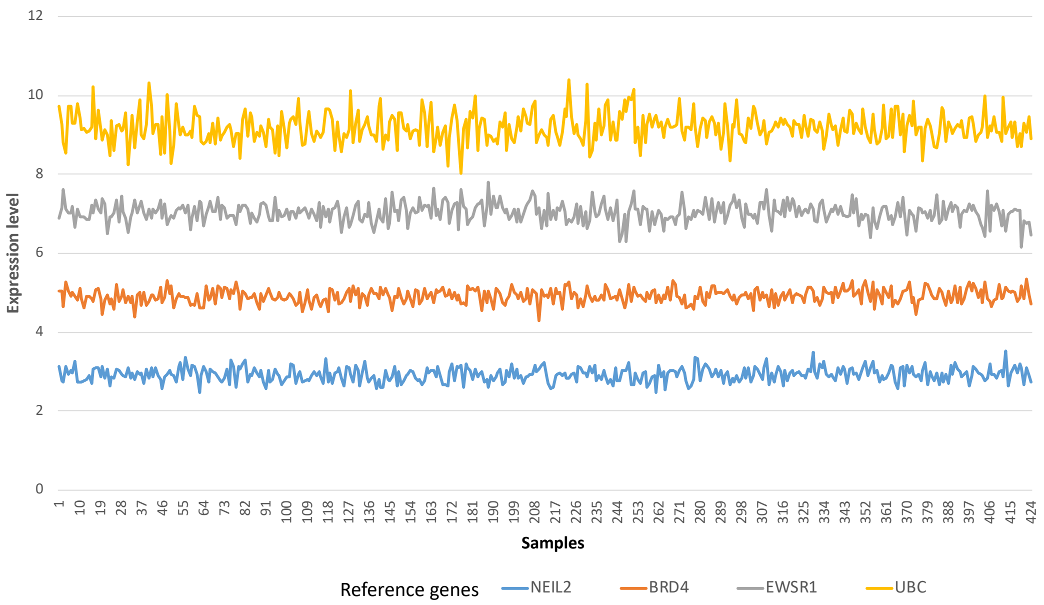

Figure S4: Expression levels of four data-driven reference genes from prostate tumor microarray samples downloaded from GEO (Accession: GSE62872).

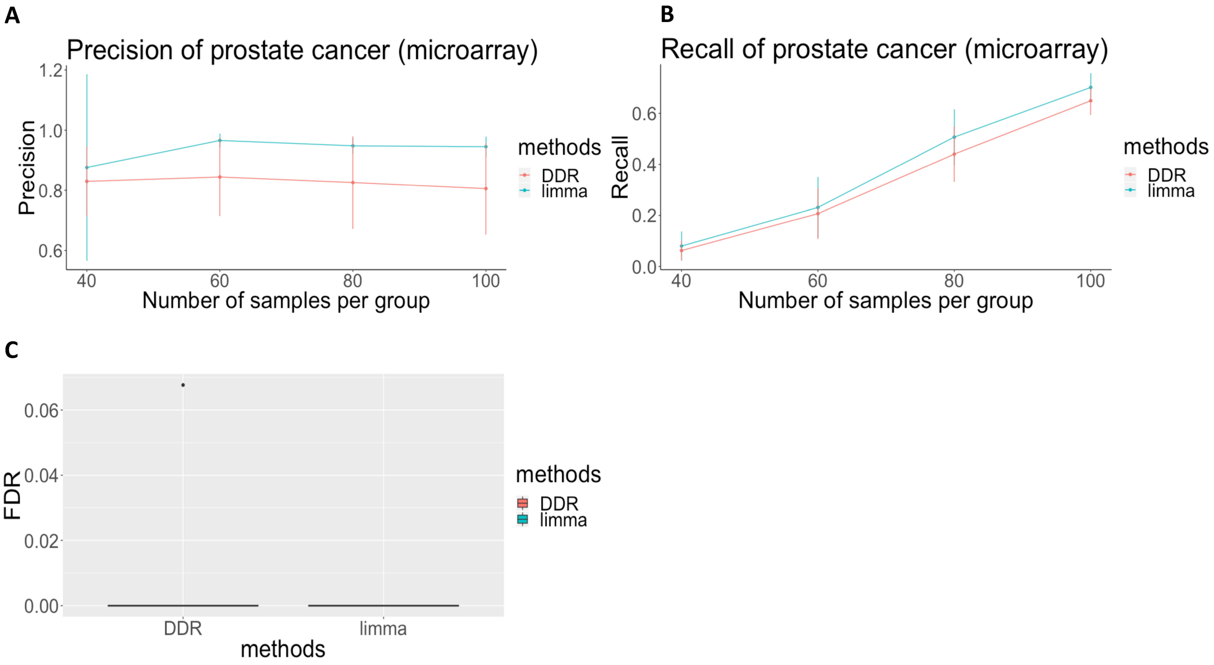

Figure S5: Precision (**A**), recall (**B**) and FPR (**C**) of methods in prostate tumor microarray dataset from GEO (Accession: GSE62872).

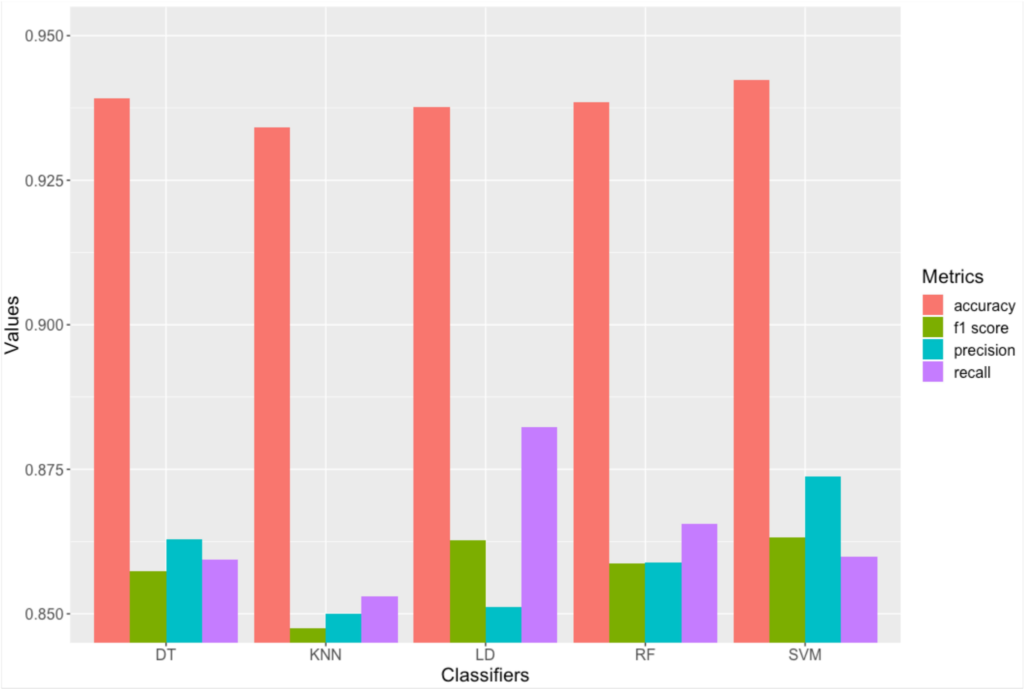

Figure S6: Classification performance different machine learning classifiers using classified tiers of four biomarkers for classifying TNBC and non-TNBC in TCGA-BRCA RNA-Seq dataset.

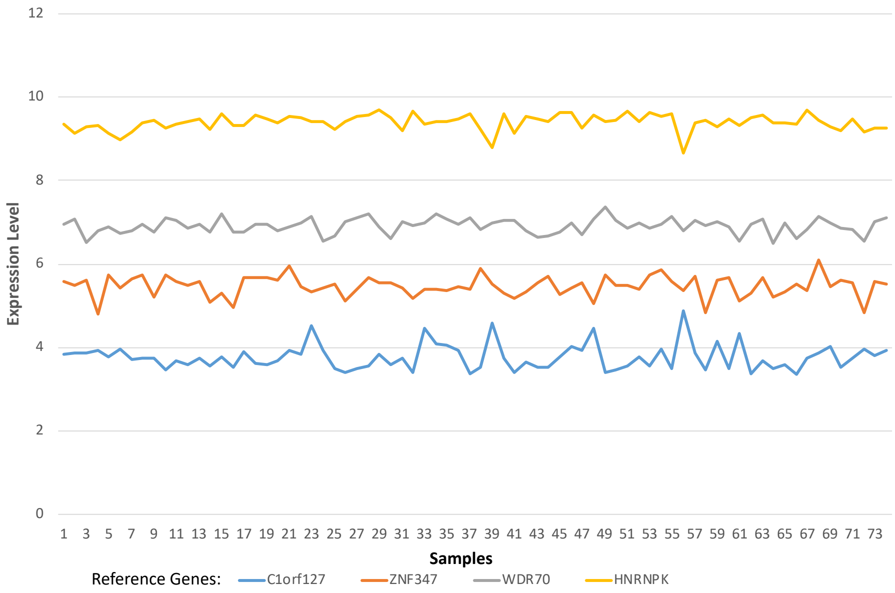

Figure S7: Expression levels of four data-driven reference genes from medulloblastoma microarray dataset from GEO (Accession: GSE37418).

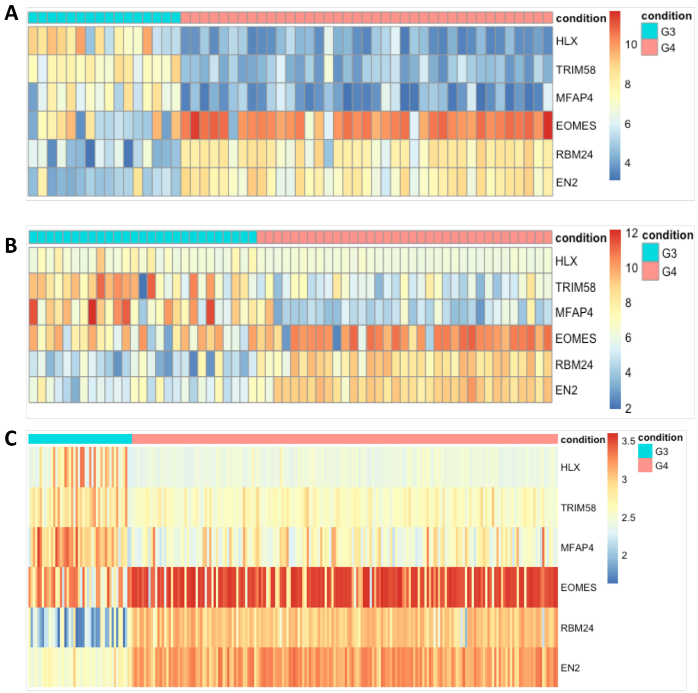

Figure S8: The expression heatmaps for signature genes between G3 (turquoise) and G4 (red) in GSE37418(**A**), GSE21140(**B**), and GSE37382(**C**).

Table S1: RNA-seq samples from Tumor-Educated Platelets

Cancer subtype	Number of samples
Breast cancer	39
Colorectal cancer	42
Glioblastoma	40
Hepatobiliary cancer	14
Lung cancer	60
Pancreatic cancer	35
Healthy contro	55

Table S2: Microarray samples for Medulloblastoma

Subtype	GSE37418	GSE21140	GSE37382
WNT	8	8	0
SHH	11	33	51
G3	16	27	46
G4	39	35	188

Table S3: Feature table for classifying TNBC and non-TNBC

BCL11A	B3GNT5	Conditions
2	2	TNBC
1	2	TNBC
2	2	TNBC
2	2	TNBC
0	0	non-TNBC
0	0	non-TNBC
0	1	non-TNBC
1	1	non-TNBC

Table S4: Overlaps of DEGs identified between the different methods in TCGA-BRCA dataset and TCGA-LUAD dataset

**Method**	DDR	EdgeR_GLM	EdgeR_EXACT	DESeq	DESeq2
TCGA-BRCA dataset					
DDR	100	87	88	48	87
EdgeR_GLM		100	99	85	85
EdgeR_EXACT			100	85	85
DESeq				100	98
DESeq2					100
TCGA-LUAD dataset					
DDR	100	80	80	52	80
EdgeR_GLM		100	99	81	92
EdgeR_EXACT			100	81	92
DESeq				100	98
DESeq2					100
